# The use of indocyanine green for lateral lymph node dissection in rectal cancer—preliminary data from an emerging procedure: a systematic review of the literature

**DOI:** 10.1007/s10151-024-02930-6

**Published:** 2024-05-18

**Authors:** D. Kehagias, C. Lampropoulos, A. Bellou, I. Kehagias

**Affiliations:** 1 Department of General Surgery, General University Hospital of Patras, University of Patras, 26504 Rio, Greece; 2 Intensive Care Unit, Saint Andrew’s General Hospital, 26335 Patras, Greece; 3 Intensive Care Unit, Department of Anesthesiology and Intensive Care Medicine, General University Hospital of Patras, 26504 Rio, Greece; 4 Department of General Surgery, General University Hospital of Patras, 26504 Rio, Greece

**Keywords:** Indocyanine green, Fluorescence, Lateral lymph node dissection, Rectal cancer, Colorectal surgery

## Abstract

**Introduction:**

Lateral lymph node dissection (LLND) for rectal cancer is still not a widely established technique owing to the existing controversy between Eastern and Western countries and the lack of well-designed studies. The risk of complications and the paucity of long-term oncological results are significant drawbacks for further applying this technique. The use of indocyanine green (ICG) near-infrared (NIR) fluorescence for LLND appears as a promising technique for enhancing postoperative and oncological outcomes. This review aims to evaluate the emerging role of ICG during LLND and present the benefits of its application.

**Materials and methods:**

Systematic electronic research was conducted in PubMed and Google Scholar using a combination of medical subject headings (MeSH). Studies presenting the use of ICG during LLND, especially in terms of harvested lymph nodes, were included and reviewed. Studies comparing LLND with ICG (LLND + ICG) or without ICG (LLND-alone) were further analyzed for the number of lymph nodes and postoperative outcomes.

**Results:**

In total, 13 studies were found eligible and analyzed for different parameters. LLND + ICG is associated with significantly increased number of harvested lateral lymph nodes (*p* < 0.05), minor blood loss, decreased operative time, and probably decreased urinary retention postoperatively compared with LLND-alone.

**Conclusions:**

The use of ICG fluorescence during LLND is a safe and feasible technique for balancing postoperative outcomes and the number of harvested lymph nodes. Well-designed studies with long-term results are required to elucidate the oncological benefits and establish this promising technique.

## Introduction

Dual lymphatic drainage of the lower rectum was discovered in 1895 when Gerota first described the upward and lateral route by injecting dye into cadavers [1]. This finding was further evaluated with more cadaveric studies, which demonstrated that lateral pelvic lymph nodes (LPLNs) are mainly distributed to the internal iliac and obturator spaces [2]. These spaces shape a triangle on the lateral side of the pelvis, whose boundaries are the external iliac artery laterally, the ureter medially, and the urinary bladder caudally [3]. On the basis of the findings of different studies, the incidence of LPLN metastasis from low rectal cancer is approximately 15% [4].

Although lateral pelvic lymph node disease (LPLND) is considered systemic in the West, in the East it is described as locoregional disease, since many studies indicate a higher rate of local recurrence after positive LPLNs [5, 6]. Total mesorectal excision (TME), as proposed by the pioneer Professor Bill Heald, together with neoadjuvant chemoradiotherapy (NCRT), is the mainstay of treatment for advanced low rectal cancer in the West [7, 8]. On the other hand, in the East according to the Japanese guidelines, TME with LLND, without NCRT is proposed for advanced T3/T4 low rectal cancers [9]. Mounting evidence from the long-term results of the randomized clinical trial JCOG012 failed to show non-inferiority in local recurrence rates after TME compared with TME and LLND alone for stage III rectal cancer, further supporting the locoregional nature of LPLNs [10].

Nevertheless, the high risk of complications and the modest oncological outcomes after LLND are a major concern, leading many surgeons to deal with skepticism about this technique [11, 12]. Blood loss, increased operative time, and urogenital dysfunction owing to inadvertent autonomic nerve injury are the main aspects requiring standardized and minimally invasive approaches. Indocyanine green (ICG) near-infrared (NIR) fluorescence is an innovative technology enabling the detection of the lymphatic drainage of rectal cancer when injected submucosally, offering the advantage of improved lymph node harvesting with greater accuracy and enhanced postoperative outcomes [13]. Furthermore, the application of ICG NIR fluorescence enables lymph node harvesting not only by directly performing a lateral pelvic dissection, but also by detecting the lateral pelvic sentinel lymph node (LPSLN) and deciding further treatment depending on its metastatic status [14]. The aim of the present literature review was to assess lymph node harvesting and postoperative outcomes after LLND with the guidance of ICG NIR fluorescence.

## Materials and methods

### Study hypothesis and endpoints

The main hypothesis of this systematic review was whether the use of ICG NIR fluorescence benefits LLND in terms of oncological and postoperative outcomes when compared with LLND without the use of ICG. The primary endpoint of this review was to evaluate the effect of ICG-guided LLND on the number of harvested lateral lymph nodes compared with LLND-alone. Secondary endpoints included assessing intraoperative parameters, such as operative time and blood loss, as well as postoperative outcomes, including length of stay and urinary retention. Additionally, the review aims to analyze the incidence of LLN metastasis detected with ICG guidance.

### Search strategy

**Fig. 1:**
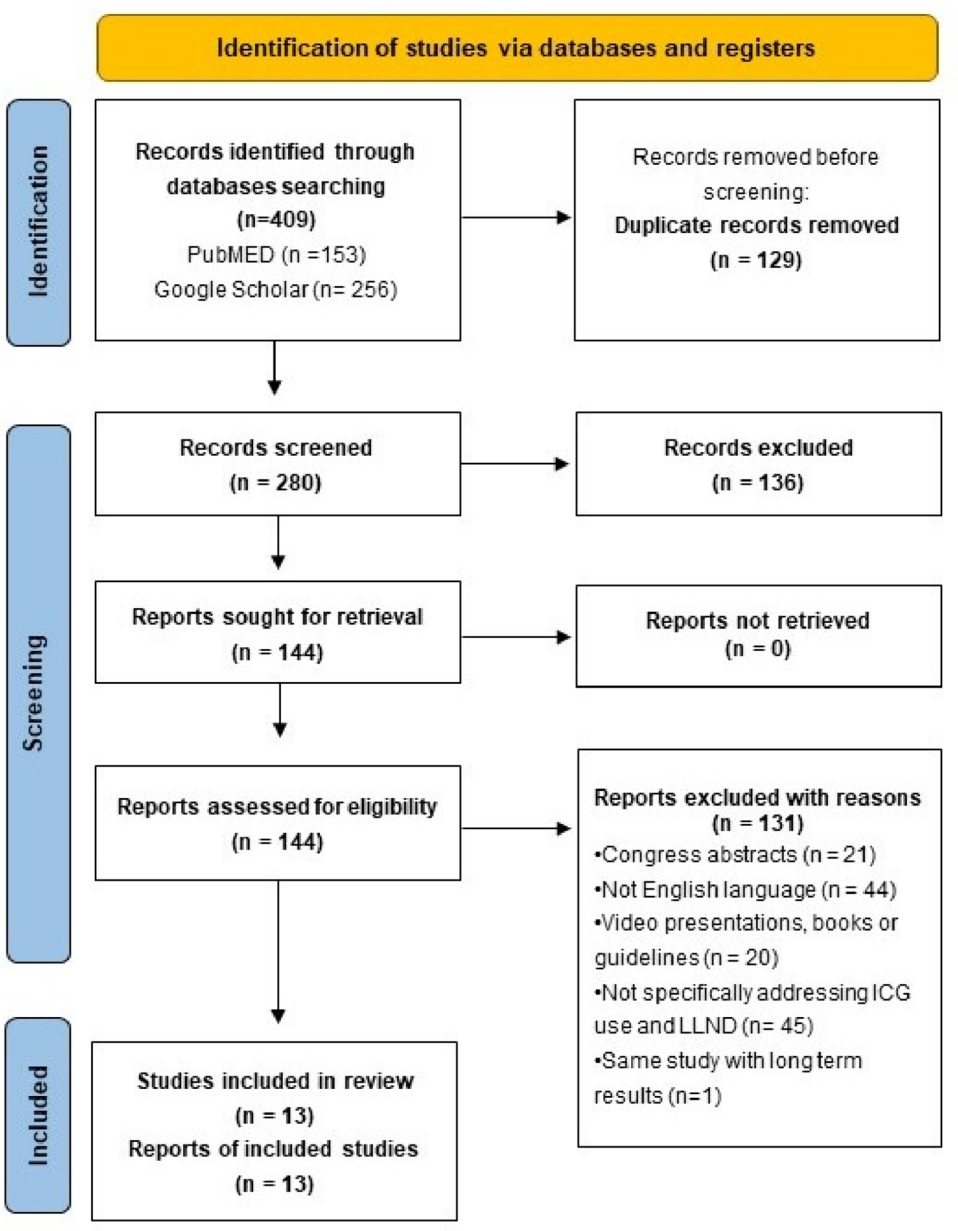
PRISMA flowchart for the retrieved and included studies

An electronic search of literature was conducted with a systematic method in the PubMed^®^ database (National Library of Medicine, Bethesda, MD, USA) and Google Scholar^®^ academic search engine from its inception until 21 November 2023. The literature search adhered to the screening guidelines for Preferred Reporting Items for Systematic Reviews and Meta-Analyses (PRISMA; Fig. 1) [15].

The search strategy included the use of the keywords “indocyanine green,” “ICG,” “lymph nodes,” “lateral lymph node dissection,” “rectal neoplasms,” and “rectal cancer” in different combinations. Besides the above keywords, the medical subject headings (MeSH) terms “indocyanine green,” “lymph nodes,” and “rectal neoplasms” were also utilized.

After the identification of the records from the databases, duplicate articles were removed. The screening process involved title and abstract identification, with irrelevant articles being excluded. Furthermore, the references of the retrieved articles were screened separately to identify additional eligible studies.

### Selection of the studies

The retrieved articles were meticulously analyzed and examined for eligibility. Original articles in English evaluating the use of ICG NIR fluorescence in LLND were deemed eligible. Case series, case reports, and comparative studies, with or without propensity matching, were included in the review. Studies that compared LLND + ICG versus LLND-alone were further evaluated to identify potential benefits of the use of ICG.

Articles in other languages, congress abstracts, video presentations, book chapters, and guidelines were excluded from the study. Studies that did not specifically address ICG use and LLND were also excluded. Studies presenting the same cohort with short- and long-term results were included once. Finally, review articles and meta-analyses were excluded from the study.

### Assessment of quality of the studies

All included studies were independently assessed by two authors for quality and risk of bias. The revised tool for the quality assessment of diagnostic accuracy studies (QUADAS-2) was applied [16]. Any discrepancies were further discussed and resolved by a third author, while the senior author supervised the whole procedure.

### Data extraction

The following data regarding patient and tumor characteristics were extracted from the reviews studied: author, year, type of study, number of patients in each study, tumor characteristics (size, distance from the dentate line, and T stage), and use of NCRT. To assess the efficacy of LLND + ICG for lymph node harvesting, data concerning the detection rate of LPLNs, the number of LPLNs, and anatomic location of metastatic LPLNs were also obtained. LLND + ICG after the detection of LPSLN was considered as a different technique and was separately analyzed. Regarding the ICG technical details, the ICG manufacturer, type of infrared camera, ICG concentration, total dosage, time, site, and route of injection were additionally extracted from the studies. For studies comparing LLND + ICG vs. LLND-alone, the number of harvested lymph nodes, operative time (min), blood loss (ml), length of hospital stay (d), incidence of urinary retention (%), and conversion rate were also extracted. Finally, for demonstrating the comparisons between the above two groups, individual data was retrieved for each parameter from the comparative studies.

## Results

### Studies and patient characteristics

**Fig. 2:**
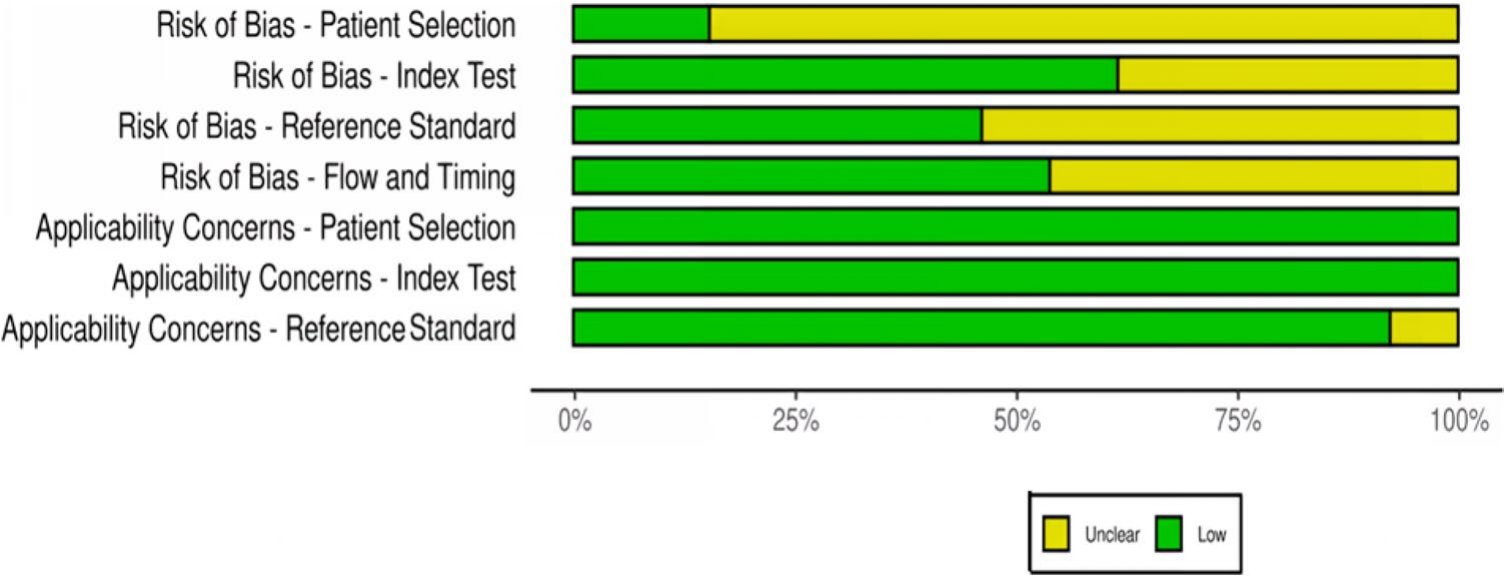
Proportion of studies with low or unclear risk of bias, %

Thirteen studies, undertaken from 2007 to 2023, were found eligible and included in the review [14, 17–28]; five of them were prospective studies, five were retrospective, and three were case reports. Results regarding the quality of the studies are depicted in Table 1 and Fig. 2. From the above studies, four compared LLND + ICG versus LLND-alone and adhered to the population, intervention, comparator, and outcome (PICO) framework for further analysis of postoperative parameters [19, 25, 27, 28] (Table 2).

**Table 1:** Results of quality assessment of the studies after using the QUADAS-2 tool

Study	Risk of bias				Applicability concerns		
	Patient selection	Index test	Reference standard	Flow and timing	Patient selection	Index test	Reference standard
Kawahara et al. [17]	?	–	–	–	–	–	–
Noura et al. [14]	?	–	?	–	–	–	–
Kazanowski et al. [18]	?	?	?	–	–	–	?
Zhou et al. [19]	–	?	–	–	–	–	–
Kim et al. [20]	?	–	?	–	–	–	–
Yasui et al. [21]	?	–	?	–	–	–	–
Bae et al. [22]	?	–	–	?	–	–	–
Sun et al. [23]	?	?	–	?	–	–	–
Zhang et al. [24]	?	–	–	?	–	–	–
Dai et al. [25]	?	–	?	?	–	–	–
Su et al. [26]	?	–	?	?	–	–	–
Watanabe et al. [27]	–	?	?	–	–	–	–
Tang et al. [28]	?	?	–	?	–	–	–

?, unclear risk; –, low risk

**Table 2:** PICO framework for intervention studies comparing LLND + ICG versus LLND-alone

Population	Patients with low rectal cancer, with or without neoadjuvant therapy
Intervention	LLND + ICG
Comparator	LLND-alone
Outcomes	Number of lymph nodes harvested (*n*), blood loss (ml), operative time (min), hospital stay (d), urinary retention (%)

A total of 220 patients with low rectal cancer underwent LLND + ICG. The tumor size and distance from the dentate line are reported in the table and expressed as mean (range), mean ± SD, and mean [interquartile range (IQR)]; most of them were located in the lower and middle rectum. The T stage was reported in 209/220 patients. Most rectal cancers were T3 (71%), followed by T2 (14%), T1 (12%), and T4 (3%). NCRT was administered in 41.3% of patients (Table 3).

**Table 3:** Studies and patient characteristics

Author	Year	Type of study	*n*	Tumor size (cm)	Tumor height from dentate line (cm)	T stage (*n*)	Neoadjuvant therapy
Kawahara et al. [17]	2007	Prospective	14	5.3 (3–7)^a^	Lower rectum	T3	None
Noura et al. [14]	2010	Prospective	25	4.5 ± 1.9^b^	3.1 ± 2.3^b^	T1 (1) T2 (5) T3 (19)	None
Kazanowski et al. [18]	2015	Prospective	5	NR	Lower rectum	NR	NCRT
Zhou et al. [19]	2019	Retrospective comparative	12	3.5 ± 1.4^b^	< 8	T1 (3) T2 (2) T3 (6) T4 (1)	NCRT (2/12)
Kim et al. [20]	2020	Prospective	10	NR	Lower rectum	T1 (1) T3 (9)	NCRT
Yasui et al. [21]	2021	Retrospective	21	4,5 (1–8)^a^	5 (1–7)^a^	T1 (7) T3 (14)	None
Bae et al. [22]	2021	Case report	1	NR	4	T4	NCRT
Sun et al. [23]	2022	Case report	1	NR	7	NR	NCRT
Zhang et al. [24]	2022	Case report	1	2.4	5	NR	None
Dai et al. [25]	2022	Retrospective comparative	20	4.1 ± 1.4^b^	< 8	T1 (4) T2 (5) T3 (9) T4 (2)	NCRT (3/20, 15%)
Su et al. [26]	2023	Retrospective	23	3.2 (1.2–6.5)^a^	4.3 (1–8)^a^	T1 (2) T2 (6) T3 (15)	NCRT (18) NCT (3) None (2)
Watanabe et al. [27]	2023	Retrospective multicentered comparative	58	3.8 (2.5–5)^c^	5 (4–6.8)^c^	T1 (4) T2(10) T3(38) T4(3)	No (23/58, 39.7%) NCT (35/58, 60.3%)
Tang et al. [28]	2023	Prospective non-randomized comparative	29	4 (*n* = 21) > 4 (*n* = 8)	4 (3–5)^c^	T1 (4) T3 (25)	NCRT (13) None (16)

*n* sample size, *NCRT* neoadjuvant chemo-radiotherapy, *NCT* neoadjuvant chemotherapy, *NR* not reported

^a^Values expressed as mean (range)

^b^Values expressed as mean ± standard deviation (SD)

^c^Values expressed as mean (interquartile range, IQR)

Two of the aforementioned studies including 46 out of 220 patients, involved the use of ICG for LLND after the detection of LPSLN and the evaluation of its metastatic status [14, 21].

### ICG injection technique

The ICG manufacturer was only reported in five studies, with DiagnoGreen Daiichi Pharm. (Tokyo, Japan) being the most commonly administered. Different devices of infrared cameras were used for the generation of fluorescence, with KARL STORZ GmbH & Co. KG (Germany) being the most operated. ICG concentration of 2.5 mg/ml and a total dosage of 1 ml were more frequently applied (39% and 54%, respectively). The preferred time point for ICG injection was immediately after anesthesia (70%), when an anoscope or endoscope was used for injecting ICG, usually at three to four points in the peritumoral submucosa (Table 4).

**Table 4:** ICG injection technique

Author	ICG manufacturer	Infrared Camera	ICG concentration (mg/ml)	ICG Total Dose (ml)	Time of injection	Site of injection	Route of injection
Kawahara et al. [17]	NR	Olympus Corp, Tokyo, Japan	5	6	0.5 h BA	SM 3 points	Transanal endoscope
Noura et al. [14]	DiagnoGreen Daiichi Pharmaceuticals, Tokyo, Japan	Photo dynamic eye, Hamamatsu, Japan	5	1	AA	SM 4 points	Transanal endoscope
Kazanowski et al. [18]	NR	PINPOINT Novadaq Corp, Ontario, Canada	5	2–5	AA	SM	Transanal anoscope
Zhou et al. [19]	Dandong Yichuang Pharmaceutical Co., Dandong, China	KARL STORZ GmbH & Co. KG, Germany	0.1	4	AA	SM 4 points	Transanal anoscope
Kim et al. [20]	NR	Firefly, DaVinci Intuitive Surgical, Inc. California, USA	2.5	1	3–5 h BA	SM 3 points	Transanal anoscope
Yasui et al. [21]	DiagnoGreen Daiichi Pharmaceuticals, Tokyo, Japan	KARL STORZ GmbH & Co. KG, Germany	5	1	AA	SM 4 points	Transanal endoscope
Bae et al. [22]	NR	Firefly, DaVinci Intuitive Surgical, Inc. California, USA	NR	1	AA	SM	NR
Sun et al. [23]	NR	KARL STORZ GmbH & Co. KG, Germany	NR	2	24 h BA	SM	Transanal anoscope
Zhang et al. [24]	NR	NR	2.5	1	AA	SM 4 points	Transanal anoscope
Dai et al. [25]	NR	KARL STORZ GmbH & Co. KG, Germany	2.5	1	AA	SM	Transanal anoscope
Su et al. [26]	Eisai Co. Ltd, Tokyo, Japan	Optocam 2100 Optomedic, Guangdong, China	2.5	1.5	AA	SM 3 points	Transanal anoscope
Watanabe et al. [27]	DiagnoGreen Daiichi Pharmaceuticals, Tokyo, Japan	KARL STORZ GmbH & Co. KG, Germany; Stryker Corporation (1588 AIM Platform; Michigan, USA)	2.5	1	AA	SM 4 points	Transanal direct vision or endoscope
Tang et al. [28]	NR	DPM-III-01 (Zhuhai Dipu Medical Technology Co., Ltd.)	1.25	0.8	1 h BA	SM 3–4 points	Transanal anoscope or colonoscope

*BA* before anesthesia, *AA* after anesthesia, *SM* submucosa, *NR* not reported

### Overall harvested lymph nodes after LLND with ICG guidance

From the eleven studies evaluating the harvested lateral lymph nodes after ICG, the overall detection rate of LPLNs after ICG use in the studies reported was 80.7% (42/52 patients). A mean number of 12.9 lateral lymph nodes per LLND was identified. The number of harvested lymph nodes was expressed as the mean (range), mean ± SD, or mean (IQR). From the data retrieved, a total of 25 LPLNs were reported as metastatic, and they were located at the internal iliac (68%) and obturator space (32%; Table 5).

**Table 5:** Lateral pelvic lymph nodes (LPLNs) detected with the use of ICG

Author	Year	*n*	Detection rate of LPLN with ICG (%)	Number of LPLNs harvested	Anatomic location of metastatic LPLNs (number)
Kawahara et al. [17]	2007	14	6/14 (43%)	16.9 (14–20)^a^	Internal iliac (6) Obturator (0)
Kazanowski et al. [18]	2015	5	5/5 (100%)	NR	Internal iliac (5)
Zhou et al. [19]	2019	12	–	11.5 ± 5.9^b^	NR
Kim et al. [20]	2020	10	10/10 (100%)	12 (6–26)^a^	Internal iliac (4, 80%) Obturator (1, 20%)
Bae et al. [22]	2021	1	–	12	NR
Sun et al. [23]	2022	1	–	14	Obturator (7) Internal iliac (2)
Zhang et al. [24]	2022	1	–	6	Left internal iliac
Dai et al. [25]	2022	20	–	19.2 ± 6.6^b^	NR
Su et al. [26]	2023	23	21/23 (76.2%)	10.2 (3–18)^a^	Most common obturator
Watanabe et al. [27]	2023	58	–	14 (10–18)^c^	NR
Tang et al. [28]	2023	29		12 (8–19)^c^	Most internal iliac

Number of harvested lymph nodes and location of metastatic nodes

*n* sample size, *NR* not reported, *LPLN* lateral pelvic lymph nodes

^a^Values expressed as mean (range)

^b^Values expressed as mean ± standard deviation (SD)

^c^Values expressed as mean (interquartile range, IQR)

### Lateral pelvic sentinel lymph node harvesting after ICG guidance

In total, 2 out of 13 studies evaluated the number of LPSLN harvested after the use of ICG. A total of 46 patients were included and the overall detection rate of lateral pelvic sentinel lymph node after ICG use was 84.7% (39/46 patients). The total number of sentinel nodes harvested in both studies was 80, while approximately two lateral sentinel lymph nodes per patient were estimated. Regarding the anatomic location of the sentinel lymph nodes, 48.7% (35/80) were located in the obturator space, 43.7% in the internal iliac space while only 7.6% (6/80) were found in the common iliac space (Table 6).

**Table 6:** Lateral pelvic sentinel lymph nodes (LPSLN) detected with the use of ICG

Author	Year	*n*	Detection rate of LPSLN with ICG (%)	Number of LPSLNs harvested	Median number of LPSLNs per patient	Anatomic location of metastatic SNs (number)
Noura et al. [14]	2010	25	23/25 (92%)	48 SN	2.1 (1–4)^a^	Internal iliac (25/48, 52%) Obturator (19/48, 40%) Common iliac (4/48, 8%)
Yasui et al. [21]	2021	21	16/21 (76.2%)	32 SN	2 (1–5)^a^	Obturator (20/32, 63%) Internal iliac (10/32, 31%) Common iliac (2/32, 6%)

Number of harvested sentinel lymph nodes and anatomic location

*n* sample size, *SN* sentinel node, *NR* not reported

^a^Values expressed as mean (interquartile range, IQR)

### Comparison of LLND + ICG versus LLND alone

Overall, four studies, three retrospective and one prospective, compared LLND + ICG versus LLND-alone in terms of lymph node harvesting, operative time, blood loss, length of hospital stay, and urinary retention. The total number of patients was divided into two groups: LLND + ICG and LLND-alone, consisting of 119 and 158 patients, respectively. All patients in each group underwent laparoscopic TME with LLND under ICG guidance, while only 4 out of 158 patients in the LLND-alone group were converted in an open approach because of intraoperative bleeding. Lateral lymph nodes harvested were significantly higher in the LLND + ICG group compared with LLND-alone in all of the studies included (*p* < 0.05). LLND + ICG was related to increased operative time in two out of four studies (*p* < 0.05), while intraoperative blood loss was significantly less in the LLND + ICG group in three out of four studies (*p* < 0.05). The length of hospital stay was shorter after LLND + ICG (*p* < 0.05) in two out of four studies. Regarding the incidence of urinary retention, an overall lower rate after LLND + ICG was observed, though without reaching statistical significance (Table 7).

**Table 7:** Comparative studies LLND + ICG versus LLND-alone

Author	Parameters	LLND + ICG (*n* = 119)	LLND-alone (*n* = 158)	*p* value
Zhou et al. [19]	*n*	12	30	
	Number of harvested LPLNs	11.5 ± 5.9^a^	7.1 ± 4.8^a^	**0.017**
	Operative time (min)	255.7 ± 65.2^a^	273.1 ± 73.3^a^	0.108
	Blood loss (ml)	55.8 ± 37.5^a^	108 ± 52.7^a^	**0.003**
	Hospital stay (d)	9.2 ± 1.6^a^	9.7 ± 2^a^	0.393
	Urinary retention, *n* (%)	0 (0)	0 (0)	
	Conversion to open, *n* (%)	0 (0)	2 (7)	
Dai et al. [25]	*n*	20	20	
	Number of harvested LPLNs	19.2 ± 6.6^a^	15 ± 4.6^a^	**0.024**
	Operative time (min)	386 ± 45^a^	332 ± 48^a^	**0.001**
	Blood loss (ml)	22 ± 9^a^	89 ± 14^a^	**< 0.001**
	Hospital stay (d)	6 ± 2.2^a^	8 ± 3.4^a^	**0.033**
	Urinary retention, *n* (%)	0 (0)	2 (10%)	0.147
	Conversion to open, *n* (%)	0 (0)	2 (10%)	
Watanabe et al. [27]	*n*	58	58	
	Number of harvested LPLNs	14 (10–18)^b^	9 (5–11)^b^	**< 0.001**
	Operative time (min)	426 (382–457)^b^	369 (324–411)^b^	**< 0.001**
	Blood loss (ml)	13 (5–125)^b^	110 (35–188)^b^	**0.001**
	Hospital stay (d)	14 (10–19)^b^	17 (13–21)^b^	**0.03**
	Urinary retention, *n* %	7 (12.1%)	13 (22.4%)	0.22
	Conversion to open, *n* (%)	0 (0)	0 (0)	
Tang et al. [28]	*n*	29	50	
	Number of harvested LPLNs	12 (8–19)^b^	9 (6–13)^b^	**0.006**
	Operative time (min)	275 (230–333)^b^	256 (210–300)^b^	0.090
	Blood loss (ml)	30 (30–100)^b^	30 (20–50)^b^	0.934
	Hospital stay (d)	5 (5–6)^b^	6 (5–8)^b^	0.147
	Urinary retention, *n* (%)	0 (0)	8 (16%)	0.059
	Conversion to open, *n* (%)	0 (0)	0 (0)	

Harvested lymph nodes, operative time, blood loss, hospital stay, and urinary retention

Bold values indicate statistical significance (*p* < 0.05)

*n* sample size, *ICG* indocyanine green, *LPLN* lateral pelvic lymph nodes, *d* days

^a^Values expressed as mean ± standard deviation (SD)

^b^Values expressed as mean (interquartile range, IQR)

## Discussion

The scope of this literature review was to provide solid and valuable information regarding the innovative application of ICG during LLND and present the potential benefits in terms of lymph node harvesting and postoperative outcomes for the patients. On the basis of the findings of the study, ICG use is associated with a significantly increased number of lateral pelvic lymph nodes harvested, while it is associated with decreased blood loss, a shorter hospital length of stay, and a lower incidence of urinary retention postoperatively.

Patients with low rectal cancer can develop LPLN metastasis located in the common iliac, internal iliac, and obturator spaces. The incidence of metastasis depends on the stage of the tumor and the distance from the anal verge. In this study, most patients presented with T3-stage tumors located mostly at the low or middle rectum. In a retrospective multicenter Japanese study involving 930 patients, the incidence of LPLN metastasis was 18.1%, with T3–T4 and low rectal cancers presenting the highest risk [29]. In another retrospective analysis, Ueno et al. showed similar results and pointed out that rectal tumors within 2 cm from the dentate line posed a 40% risk of LPLN metastasis [30]. Whether LPLND should be considered a metastatic disease or a locoregional one that can be surgically controlled is still controversial, leading to different treatment approaches [6]. In Japan, it is considered a regional disease, and it is controlled with LLND instead of NCRT. From the long-term results of the randomized trial JCOG012, the 7-year lateral local recurrence-free survival rate was 85.3% in the TME + LLND group and 80.3% in the TME-alone group, the difference was not statistically significant. Nevertheless, the cumulative local recurrence rate was significantly lower in the TME + LLND group, with the only different pattern of recurrence being in the lateral pelvis, enhancing the fact that LPLND is locoregional [10]. However, more well-designed studies with long-term results are needed to assess the effect of LLND, with or without NCRT, in terms of local recurrence.

The use of ICG during LLND is an auspicious technique since it provides great accuracy and efficacy for identifying lymphatic tissue inside the deep and narrow pelvis, leading to an increased number of lymph nodes harvested and probably improved oncological outcomes [13]. Various applications of ICG use are described in literature, from sentinel node identification to the recognition and matching of suspicious lymph nodes after 3D reconstruction images [20]. In this way, unnecessary LLND can be avoided, resulting in less morbidity, while the status of the sentinel lymph node can be used as a strong predictive factor necessitating the need for further interventions. The identification of LPSLN with ICG guidance was described in two of the included studies, determining the need of LLND after assessment of the sentinel node’s metastatic status with hematoxylin–eosin, providing a valuable tool in the arsenal of the surgeon and avoiding a perhaps unnecessary LLND [14, 21]. On the basis of the findings of the literature review, ICG has a detection rate of 80.7% for lateral lymph nodes and 84.7% for LPSLN. A meta-analysis including 248 patients investigated the efficacy of ICG in sentinel lymph node detection and showed a sensitivity and specificity of 73.7 and 100.0, respectively, concluding that ICG is a reliable method for lymph node detection [12]. Furthermore, study analysis showed that ICG leads to an increased number of lymph nodes harvested in all of the comparative studies, which may be interpreted as an important oncological benefit. However, it is crucial to underline that the presence of ICG in a lymph node does not guarantee its metastatic status. Hence, the translation of harvesting a greater number of lateral lymph nodes with ICG into a direct oncologic benefit demands further investigation. Regarding the long-term outcomes of ICG use, only one retrospective study from Watanabe et al. reports that after 3 years, LLND + ICG is associated with a significantly lower local recurrence rate compared with LLND-alone (0% versus 9.3%, *p* = 0.048) [27]. Therefore, even though the high detection rate and the increased number of harvested lymph nodes are valuable assets of ICG use, these have to be cautiously interpreted in terms of local recurrence and overall disease-free survival.

Furthermore, it is important to note that the potential oncological benefits of LLND need to be balanced against the postoperative outcomes. On the basis of the findings of many studies, LLND performed by an experienced surgeon is associated with increased operative time, increased blood loss, and a higher rate of urogenital complications and groin pain owing to inadvertent injuries to the autonomic plexus and the obturator nerve, respectively [11, 31]. From the studies comparing LLND with or without ICG guidance, ICG use was strongly associated with decreased blood loss and perhaps increased operative time with a shorter length of hospital stay. In these comparative studies the offered surgical approach was minimally invasive with laparoscopic TME and LLND through ICG guidance. Only 4 out of 158 patients in the LLND-alone group were converted in an open approach because of intraoperative bleeding. Thus, the reduced blood loss in the LLND + ICG group was related to the meticulous dissection of lymph nodes through ICG guidance and was not a result of the surgical approach, while ICG guidance during LLND might also facilitate the procedure by reducing the conversion rate. Besides these findings, the incidence of urinary retention was higher in the LLND-alone group, suggesting that ICG use during LLND is a safe technique respecting not only the anatomical structures but also the oncological outcomes. Moreover, Tang et al., in a prospective study, showed that LLND + ICG not only increases the number of lymph nodes harvested, but also that the application of simultaneous real-time ICG angiography ensures the preservation of the inferior vesical artery, leading to improved postoperative urinary function. Another important factor that may influence the results of LLND + ICG in terms of postoperative outcomes is NCRT, as it is associated with difficulty in dissection and increased nerve injury [32]. Thus, the application of neoadjuvant treatment should always be taken into consideration in studies evaluating complications after LLND with or without ICG use.

ICG NIR fluorescence is a feasible and reproducible method, even though various technical details regarding dosage, concentration, site, and time of injection are described. Most studies adopt a technique in which ICG is injected after anesthesia at 3–4 points in the submucosa with the use of an endoscope or anoscope. Submucosal injection has the highest sensitivity since the lymphatic network is located in the submucosal layer [33]. Hence, the existing heterogeneity of the ICG injection technique creates an essential problem for determining the accuracy of this method. Moreover, the optimal timing for ICG injection is still arguable, and more well-designed studies are needed to standardize this technique.

Whether LPLND is considered systemic or locoregional, as well as the controversy between the West and East, have led to a limited number of well-designed studies evaluating the technique of LLND in terms of oncological and postoperative outcomes. On the one hand, the West has still not adopted LLND, and on the other hand, the East tries to standardize the technique and optimize their results with minimally invasive methods, like ICG guidance. This created a strong limitation for this literature review, as the studies included were mainly retrospective, with heterogeneity in the study population and the technique of ICG injection, resulting in unclear risk of bias, preventing the performance of a systematic meta-analysis. Furthermore, the data presented in this systematic review are only preliminary, since only a limited number of studies investigating this technique have been published and the sample size is limited as well, leading to many confounding factors, necessitating a cautious interpretation of these findings. The need for more well-designed studies with long-term results is imperative to better determine the effects of the promising LLND + ICG technique. Nevertheless, as the controversy persists, it will be a major drawback for assessing and evolving LLND with the use of ICG guidance.

## Conclusions

ICG NIR fluorescence guidance for LLND is a feasible technique, quite promising in terms of harvested lymph node number, with respectable postoperative outcomes. These findings need to be further translated in terms of oncological benefits by conducting well-designed studies and providing long-term results to establish this promising technique. Harnessing the findings of more well-designed studies, regarding the use of ICG during LLND, safer and more accurate conclusions will be drawn.

## Data Availability

The data that support the findings of this study are available on request from the corresponding author.
